# Resolving the role of femtosecond heated electrons in ultrafast spin dynamics

**DOI:** 10.1038/srep03980

**Published:** 2014-02-05

**Authors:** J. Mendil, P. Nieves, O. Chubykalo-Fesenko, J. Walowski, T. Santos, S. Pisana, M. Münzenberg

**Affiliations:** 1 I. Physikalisches Institut, Universität Göttingen, Friedrich-Hund Platz 1, 37077 Göttingen, Germany; 2 Instituto de Ciencia de Materiales de Madrid, CSIC, Cantoblanco, 28049 Madrid, Spain; 3 San Jose Research Center, HGST, a Western Digital Company, 3403 Yerba Buena Rd., San Jose, California 95135, USA; 4 Institut für Physik, Universität Greifswald Felix-Hausdorff-Straβe 6, 17489 Greifswald, Germany

## Abstract

Magnetization manipulation is essential for basic research and applications. A fundamental question is, how fast can the magnetization be reversed in nanoscale magnetic storage media. When subject to an ultrafast laser pulse, the speed of the magnetization dynamics depends on the nature of the energy transfer pathway. The order of the spin system can be effectively influenced through spin-flip processes mediated by hot electrons. It has been predicted that as electrons drive spins into the regime close to almost total demagnetization, characterized by a loss of ferromagnetic correlations near criticality, a second slower demagnetization process takes place after the initial fast drop of magnetization. By studying FePt, we unravel the fundamental role of the electronic structure. As the ferromagnet Fe becomes more noble in the FePt compound, the electronic structure is changed and the density of states around the Fermi level is reduced, thereby driving the spin correlations into the limit of critical fluctuations. We demonstrate the impact of the electrons and the ferromagnetic interactions, which allows a general insight into the mechanisms of spin dynamics when the ferromagnetic state is highly excited, and identifies possible recording speed limits in heat-assisted magnetization reversal.

The FePt L1_0_ alloy represents the most important material for novel concepts in magnetic recording due to its high magnetic anisotropy, which ensures long-time thermal stability of nanometer sized bits^1^. Thin films of FePt with perpendicular anisotropy and small grain sizes are the most promising candidates for heat-assisted magnetic recording, which could reach storage densities beyond 1 Tb/inch^2^. Patterning continuous FePt into individual bits^2^ can in principle extend recording densities to 100 Tb/inch^2^. The ultimate magnetic recording applications will also require faster bit switching and a deeper insight into the processes involved. However, non-deterministic fractioning in ultrafast magnetization reversal can limit the switching speed in recording schemes, and thus has inspired fundamental research for more than a decade^3^,^4^. Recently, a new concept of ultrafast all-optical magnetic recording with an unprecedented switching timescale below 1 ps was suggested^5^. This opened new possibilities to reduce the speed limit established by the spin-orbit coupling timescale to that governed by the much stronger exchange interaction. Here we show that in FePt the intrinsic dynamics surprisingly is connected with the electronic structure: the alloying of Pt, because of its low lying d-bands, reduces the density of states significantly^6^,^7^ and increases the electron temperature significantly above the Curie temperature. We identify the large electron energies of the heated electron system in FePt L1_0_, arising from its reduced density of states at around the Fermi level, as its origin driving the spin fluctuations^8^,^9^.

Even though CoPt_3_ was among the first thin film systems investigated^10^ since the discovery of ultrafast demagnetization in 1996 by Beaurepaire et al.^11^, most investigations were centered on samples with in-plane anisotropy. So little is known about the behavior of materials with high perpendicular anisotropy^12^,^13^,^14^. A notable exception of materials with perpendicular anisotropy is the ferrimagnetic CoFeGd, which was studied in all-optical ultrafast switching triggered by a single laser pulse^5^,^15^. The modeling of this mechanism involves the exchange interaction of the two spin subsystems^16^. Different models have been proposed^17^,^18^,^19^,^20^,^21^. On the other hand, recent work of Koopmans et al.^22^ suggests the classification of materials as “fast” (or type I) and “slow” (or type II) based on the ratio *T_C_*/*μ_at_*, where *μ_at_* is the magnetic momentum per atom and *T_C_* is the Curie temperature. In both cases, there is an initial sub-picosecond fast demagnetization. However, in the first case the fast femtosecond demagnetization is followed by a magnetization recovery (as in Ni^23^), while in the latter a second slower demagnetization takes place. The recovery occurs on the timescale on the order of 50 ps and more (as in Gd^24^). According to this classification FePt should be regarded as a fast magnetic material. However, more recently it has been shown that in Ni both behaviors can be observed^25^. Thus, the question of whether the high anisotropy material FePt can behave as “fast” or “slow” under specific laser excitation is an open question. In addition, for thin films and granular media, the contributions of spin currents to the ultrafast demagnetization dynamics cannot be neglected^26^,^27^,^28^ that can be directly detected by the emission of THz radiation^29^. We use insulating substrates and cap layers to minimize these effects. A transition from type I to type II demagnetization is found, triggered by the laser fluence. Theoretically this is analyzed by mapping the properties of the electron system onto a Heisenberg-like spin system in a thermal ensemble that determines the timescales of the ultrafast fluctuations^30^. A coupling parameter *λ*, determined by the microscopic spin-flip transitions, couples them^31^. Via two scenarios, we calculate a different response of the electronic system to the laser pulse. We can pinpoint that the quasi-equilibrium values of the electron temperature above the Curie temperature is determining the response of the spin system. This is leading to the critical fluctuations and a slowing down, a fundamental limit to recording speeds in heat-assisted reversal on the ultrafast timescales. To enable progress in high-speed and high-capacity magnetic storage devices, a fundamental understanding of these dynamic processes is required in the future.

## Results

**Figure 1:**
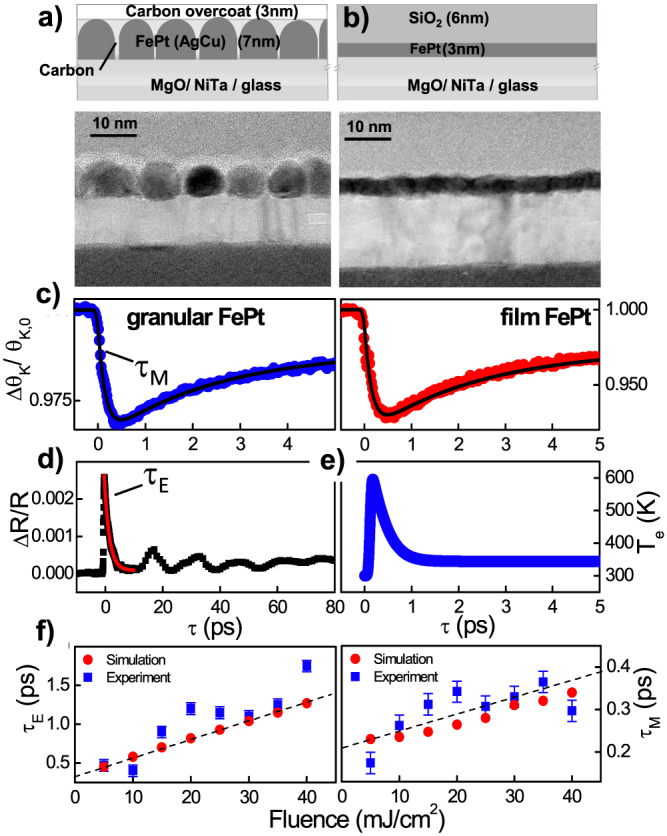
Sample characteristics: schematics and sample structure of the granular FePt recording media (a) and thin film (b) as measured by transmission electron microscopy (TEM). (c) Ultrafast magnetization dynamics for both cases after femtosecond laser excitation for a small demagnetization. Solid line: analytical three temperature model to obtain *τ_M_*. Both curves can be described with sets of identical parameters. (d) The reflectivity dynamics from which the exponential decay *τ_E_* and (e) the electron temperature *T_e_* are obtained (laser fluence 5 mJ/cm^2^). (f) Relaxation time *τ_E_* for the electron temperature and *τ_M_* for the ultrafast demagnetization is given as function of the pump fluence. The dashed line marks the linear increase.

We have studied a 3 nm-thick continuous FePt thin layer and a 7 nm-thick AgCuFePt-C granular recording media, shown in Fig. 1 a) and b). In the granular media carbon intercalates the magnetic 5–8 nm grains to separate them magnetically^32^,^33^. In Fig. 1 c) the time-resolved magneto-optical Kerr effect (Δ*θ_K_*/*θ_K_*_,0_) is shown for the granular FePt recording medium as well as the data for the continuous FePt layer for a small demagnetization of below 3–7 % *M_S_*, which was set to +/− *M_S_* beforehand. Besides the absolute scale, both can be described using identical parameters of the analytical solution of a rate equation model, shown as a continuous line: the microscopic mechanisms on the nanometer length scale dominate the dynamics on a femtosecond timescale. This leads to identical spectra for the continuous film and granular media. Moreover for the case of the recording medium having granular structure, the larger *H_S_* and different *K_u_* does not alter the magnetization dynamics. The energy scale of the magnetic anisotropy *K_u_* < 1 meV is too small to affect the dynamics on the timescale related to the energy scale of the exchange interaction. Simultaneously to the magneto-optical Kerr rotation of the probe beam, the time-resolved reflectivity is determined. The decay of the reflectivity signal is fitted to an exponential function before characteristic stress waves set in (Fig. 1 d)), according to *R*(*t*) ~ exp (−*t*/*τ_E_*). The results are presented in Fig. 1 f) showing the evolution of the characteristic timescale *τ_E_* that represents the relaxation time of the electron temperature. Experimentally, the value for *τ_E_* ~ 1 ps is a typical value expected for transition metals and consistent with previous results^35^. While for oxides discrepancies of the Kerr rotation and the genuine magnetization can be found even on long timescales^36^, this is meanwhile accepted not to be the case for metals for time scales longer than the electron equilibration time (about 50–100 fs).

**Figure 2:**
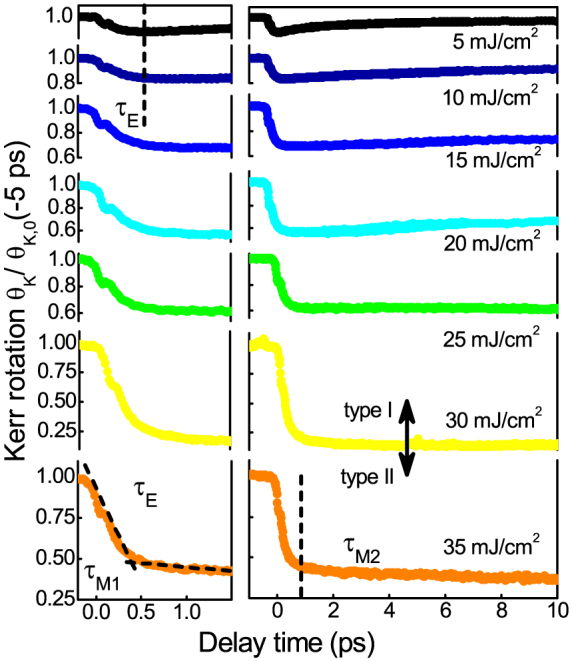
Ultrafast demagnetization dynamics of FePt: spin dynamics measured by the Kerr set-up at increasing laser fluence in steps of 5 mJ/cm^2^ from 5 mJ/cm^2^ (upper curve) to 35 mJ/cm^2^ (lower curve). The detail on the femtosecond timescale is shown with expanded scale on the left side. For the highest fluence the demagnetization still slowly progesses after the first, fast demagnetization is observed (type II material). Data is normalized to the magnetization value before the pump pulse arrives (negative delay).

For the continuous FePt layer, using moderate B-fields of 200 mT saturates the magnetization and we can extract the microscopic parameters of the ultrafast magnetization dynamics of FePt in polar geometry for a strong demagnetization. In the experiment, the fluence of the pump beam is varied from 5 to 35 mJ/cm^2^. The Kerr rotation is extracted from that of opposite external field direction^34^,^37^ to get the absolute degree of demagnetization, presented in Fig. 2. The first rapid demagnetization, *τ_M_*, occurs at a timescale of 0.15–0.38 ps, increasing with the laser pump fluence. A “kink” is observed here on the short timescales at zero delay known from preceding experiments, for example by Bigot et al.^38^. The remagnetization slows down as a function of the incident fluence, until finally a second, slower demagnetization with the absence of recovery is found above a fluence of 30 mJ/cm^2^. Thus, a transition from type I to type II as classified in Ref. 22 is observed for FePt.

**Figure 3:**
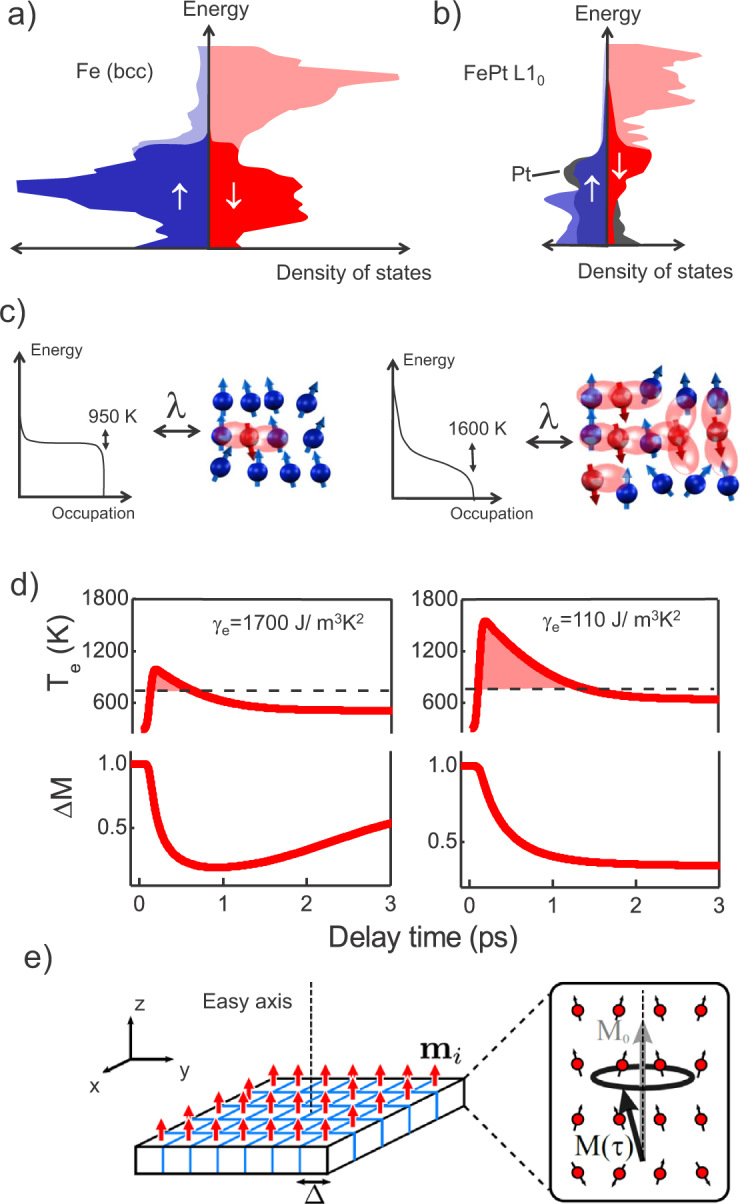
Density of states of Fe in (a) and FePt L1_0_ compound in (b): Pt alloying reduces the density of states at the Fermi level by lower lying d-bands of Pt^6^,^7^. By blue, red and grey are Fe majority, Fe minority and Pt states are given respectively. In darker color the occupied states are given. (c) Illustration of the microscopic interaction of the heated electrons *T_e_* and the spin system via spin flips coupled by *λ* for both cases. (d) Ultrafast dynamics for a hypothetical material having a large density of states and FePt using a small density of states (related to *γ_e_*) for a high laser fluence. In both cases the same reflectivity dynamics (*τ_E_*) and maximum demagnetization is calculated, but the resulting ultrafast magnetization dynamics is different. (e) Implementation of the micromagnetic Landau-Lifshitz-Bloch (LLB) model: the magnetization is described by the average over 900 thermal macrospins with a lateral cubic discretization of Δ = 3 nm with periodic boundary conditions. Each cell represents the thermodynamic average over atomistic magnetic moments (shown schematically on the right side). The thermal ultrafast heating is taken into account within each single cell by the longitudinal relaxation *M*(*τ*) and the individual macrospin allows a rotation. The lateral discretization allows the implementation of more general lateral inhomogeneous excitation (e.g. temperature gradients).

To understand the behavior, we model the ultrafast magnetization dynamics under external laser excitation by a thermal process of electronic origin^23^. The model considers that within a timescale of the order of 10 fs the electrons are thermalized and can be described by a quasi-equilibrium electron temperature *T* = *T_e_*(*t*), coupled to the spin system via the the fluctuation-dissipation theorem. We implemented the modeling within a stochastic micromagnetic Landau-Lifshitz-Bloch (LLB) model. The LLB equation for the spin S represents a thermodynamic extension of the Landau-Lifshitz-Gilbert (LLG) equation and has been derived by modeling distribution functions for thermal ensembles^39^. The thermal bath is the electron temperature *T* = *T_e_*(*t*), and the coupling-to-the bath parameter *λ* is related to microscopic scattering mechanisms. For the spin quantum number S = 1/2 this versatile equation is equivalent to the microscopic three temperature model (M3TM) proposed for the description of ultrafast processes^35^. Using the LLB with spin *S* = 3/2 it has been shown by comparison to an atomistic model for FePt that this describes its thermal equilibrium properties, e.g. damping and magnetization versus temperature, as well as the ultrafast magnetization dynamics^30^,^40^. Note that in the classical case, e.g. for S → ∞, the LLB equation can be derived as a coarse-grained approach from a classical Fokker-Planck equation for a thermal ensemble of spins, whose individual motion follows the Landau-Lifshitz-Gilbert equation, coupled to a thermal source via stochastic fluctuations in a similar way. The LLB's advantage, as compared to atomistic simulations, is that it is far less computationally intensive and thus represents a thermodynamically consistent extension of classical micromagnetism. Therefore, to extend its capabilities describing lateral inhomogenities (e.g. lateral thermal gradients or domains), here the LLB is extended to a thermal micromagnetic LLB. Combining individual thermal macrospins in a discrete array allows to model systems up to the micron-size. Thereby, each cell represents a thermodynamic average over atomistic magnetic moments. Thus, thermal ultrafast heating is taken into account within each single cell by the longitudinal relaxation, equal to *τ_M_*. However, this micromagnetic LLB allows describing locally a different thermal demagnetization, as well as a rotation against each other. In the model 900 thermal macrospins with a lateral cubic discretization of Δ = 3 nm with periodic boundary conditions are combined (Fig. 3). In detail, every single macrospin within the LLB formalism **m***_i_* = **M***_i_*/*M_e_*(0) is described by the following formalism derived by Garanin^39^,^41^,^42^: 
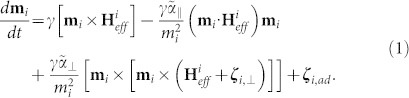
**M***_i_* is the magnetization (thermal average of atomistic spins over the volume *V* at temperature *T*), *M_e_*(*T*) is its thermal equilibrium value and *M_e_*(0) is the maximum at *T* = 0 K. The temperature dependence of *M_e_*(*T*) is evaluated via the Brillouin function with spin quantum number *S* = 3/2^43^. The effective field 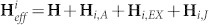 is comprised of applied, anisotropy, micromagnetic exchange, and internal exchange fields. The micromagnetic anisotropy field 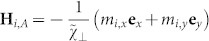 is determined by the perpendicular susceptibility 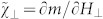. Experimental^44^ and theoretical^45^ results report that in FePt the anisotropy K scales with magnetization as ~ *m*(*T*)^2.1^. Thus, we use 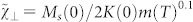. The micromagnetic exchange field is defined as^30^

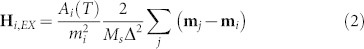
where *j* goes over neighboring elements and *A_i_*(*T*) is the micromagnetic exchange stiffness. For FePt, we derived its temperature dependence from atomistic calculations using a FePt Hamiltonian with parameters taken from local spin density approximation (LSDA)^46^. It scales with the magnetization as ~ *m*(*T*)^1.76^. Here we use 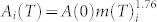, where *A*(0) = 2.2 · 10^−6^ erg/cm. The internal exchange field **H***_i_*_,*J*_ results from the thermal average of atomistic spins. At low temperatures, it is responsible for keeping the magnetization magnitude constant. At the same time this term is responsible for the critical behavior approaching ~ *T_C_*, which can be seen in the following expression: 
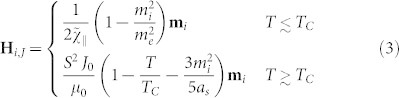
In this expression *a_s_* = 2(*S* + 1)^2^/([*S* +1]^2^ + *S*^2^) and 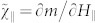 represents the longitudinal susceptibility. The latter follows from 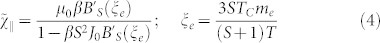
where *B*′() stands for the derivative of the Brillouin function. The relationship between the internal exchange parameter *J*_0_ (also related to *A*(*T* = 0 K), see Ref. 46) and *T_C_* is given by *T_C_* = *S*(*S* + 1)*J*_0_/3*k_B_* where *k_B_* is the Boltzmann's constant. The stochastic fields ***ζ****_i_*_,⊥_ and ***ζ****_i,ad_* are given by^47^

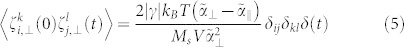

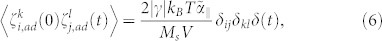
where *i* and *j* denote the macrospin number and *k* and *l* denote its Cartesian components *x*, *y*, and *z*. Finally, the longitudinal and transverse relaxation parameters are^42^

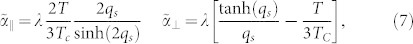
where *λ* is the microscopic relaxation parameter that couples the spin dynamics to the electron temperature *T* = *T_e_*(*t*), defined by the microscopic spin scattering rate *λ*, and *q_s_* = 3*T_C_m_e_*/[2(*S* + 1)*T*].

Via microscopic spin scattering rate *λ* and the relaxation dynamics, the magnetization dynamics is coupled to the electron temperature *T* = *T_e_*(*t*) as described above. However in turn, the electron temperature within the two temperature model (2T) is coupled to the lattice temperature *T_ph_*(*t*) via rate equations, which determine its evolution with time *t* after the pump pulse arrived: 
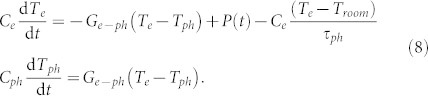
Here *C_e_* and *C_ph_* denote the specific heat of the electrons and the lattice, respectively, *G_e_*_–*ph*_ is the coupling constant determining the energy exchange between the electron and lattice systems, and *τ_ph_* = 340 ps is the heat diffusion time to the substrate. For *C_e_*, the free electron approximation is used resulting in *C_e_* = *γ_e_T_e_*, where *γ_e_* is determined by the electron density of states. *C_ph_* is set constant, since FePt has a Debye temperature well below the room temperature *T_room_*. The laser absorbed power is defined by *P*(*t*) = *I*_0_*F* exp [−(*t*/*τ_p_*)^2^] proportional to the laser pump fluence *F*. The time-resolved reflectivity reveals that the change of electron temperature depends linearly on the change in reflectivity^48^. Thus, we assume that the lacking parameters of the 2T model can be extracted from the experimental data. The thermal diffusion timescale to the substrate *τ_ph_* is obtained from the long-term magnetization behavior. The proportionality constant *I*_0_ is estimated by fitting of the experimental demagnetization value at 30 ps and the coupling-to-the bath parameter *λ* via matching the maximum demagnetization value. The microscopic parameter *λ* is related to the spin-flip probability. Assuming that the main spin-flip scattering mechanism is the same that is responsible for the energy dissipation processes on a longer timescale, *λ* is connected to the macroscopic Gilbert damping.We used 0.01 to 0.1 as a starting point for FePt^30^,^49^,^50^,^51^. This value scales with the spin-orbit interaction^51^. But generally the values of the Gilbert damping are found to be larger than the electronic coupling-to-the bath parameter *λ*. Additional contributions to the Gilbert damping are magnon-magnon scattering and inhomogeneous line broadening of the ferromagnetic resonance^34^,^52^.

The determination of the material specific constants such as *γ_e_* and *G_e–ph_* is crucial for a proper simulation of the demagnetization process. Two approaches were performed, resulting in a high and a low electron temperature profile. To model the profile for the FePt, first *G_e–ph_* = 1.5 · 10^17^ W/m^3^K is assumed in the range of Cu, Mo, and Pt^53^. Then, the analysis of the reflectivity relaxation rate gives *γ_e_* = 110 J/m^3^K^2^. This value is of the order reported for Au and Cu^53^, but is much smaller than the corresponding value for Ni and hence produces a high electron temperature^23^. In the second case of a hypothetical material, a coupling constant *G_e–ph_* = 1.8 · 10^18^ W/m^3^K, similar to Ni^23^, is assumed which gives *γ_e_* = 1700 J/m^3^K^2^. This *γ_e_* is the proper value for transition metals with a large density of states at the Fermi level due to crossing d-bands. As a consequence, a lower electron temperature is reached (Fig. 3 d)).

## Discussion

**Figure 4:**
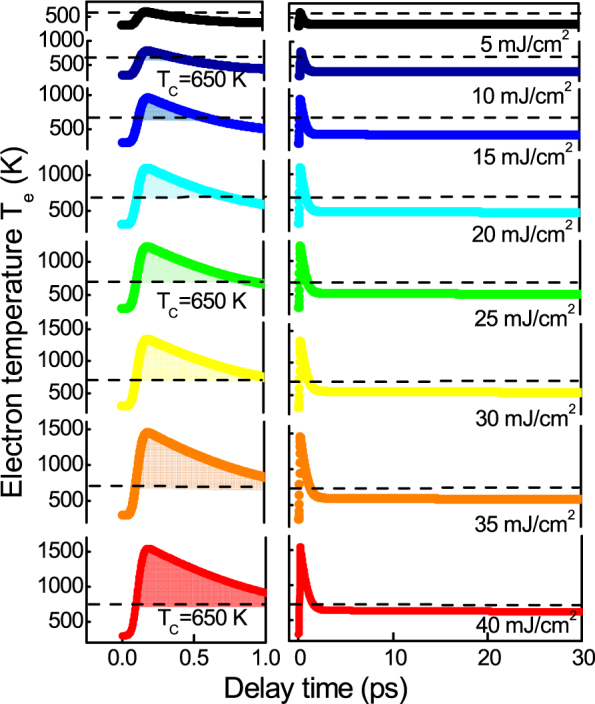
Simulation of the electron temperature *T_e_*, shown as a function of the laser pump fluence (from 5 mJ/cm^2^ (upper curve) to 40 mJ/cm^2^ (lower curve), in steps of 5 mJ/cm^2^. The 2T model is based on the set of parameters presented in Table I. The parameters are extracted from the reflectivity dynamics (Fig. 1). Within the shaded area marked in the left panel, the electron temperature exceeds the Curie temperature (dashed line).

**Figure 5:**
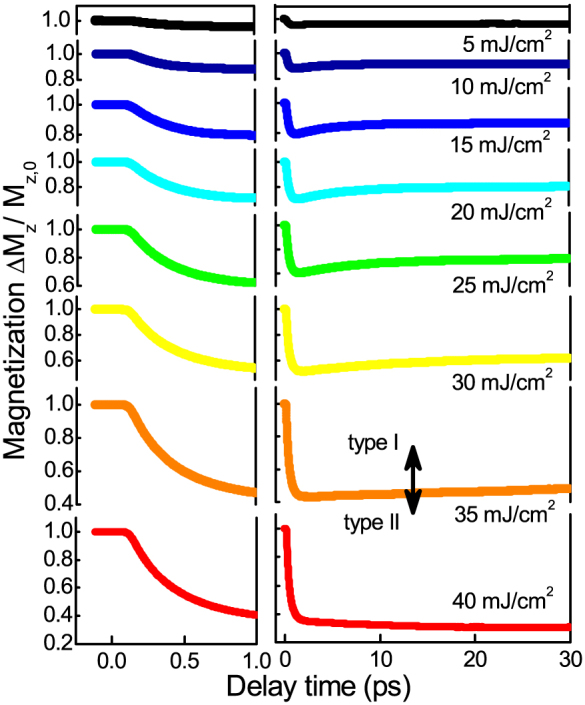
Ultrafast demagnetization dynamics obtained by integration of the LLB micromagnetic model coupled to the 2T model (Fig. 4). The model data are shown with increased laser fluence from top to bottom in steps of 5 mJ/cm^2^ from 5 mJ/cm^2^ (upper curve) to 40 mJ/cm^2^ (lower curve). The detailed view on the femtosecond timescale is shown with expanded scale on the left side.

The results for the integration of the set of the LLB equations, coupled to the 2T model, with the set of parameters for FePt are presented in Fig. 5 for all fluences. As in the experiment, the simulations show a transition between type I and type II behavior. A fair agreement is found for the evolution of the demagnetization values Δ*M*/*M*(300 *K*) = 0.05–0.7. The demagnetization time *τ_M_* are matched, and range in between *τ_M_* = 0.2–0.35 ps and, within this fluence range, they show an increase (Fig. 1 f)). Because of the large electron temperatures, a small *λ* = 0.01 is sufficient to reach the demagnetization values. Contrary to this, in case of the integration of the LLB equation coupled to the 2T model with *γ_e_* = 1700 J/m^3^K^2^ for the hypothetical material, the demagnetization time *τ_M_* stays at a constant value and is always followed by the remagnetization within several picoseconds (see Supplementary Materials). Here a large *λ* = 0.1 is necessary to reach the experimental demagnetization values. The difference can be seen in Fig. 3 d) where both scenarios are presented directly opposed to each other. We obsereve that the second scenario does not produce a transition between type I and type II behavior. The origin of the high electron temperatures is the reduced density of states of FePt by Pt alloying. Because of lower lying d-bands in Pt as compared to Fe (Fig. 3 a, b), the ferromagnet Fe becomes more noble in the FePt L1_0_ compound^6^,^7^. This is the underlying reason for the electron specific heat being more similar to Au and Cu with a small *γ_e_*. For the hypothetical scenario with large *γ_e_*, only below 1 ps a *T_e_* above the Curie temperature *T_C_* is found. For the full calculation for all fluences refer to the Supplementary Material. We conclude that the transition from type I to type II demagnetization is defined by a critical temperature that the electrons have reached. The microscopic picture behind is that the electron system drives via spin-flips the magnetic system into a region, where the ferromagnetic correlations are strongly disturbed as schematically shown in Fig. 3 c). The role of the coupling parameter *λ* is only to determine the rate of spin-flips^31^. Microscopic spin fluctuations are responsible for the decrease of the micromagnetic exchange stiffness *A*(*T*) and thermal behavior of *M_e_*(*T*) within the LLB model. The crucial effect, however, is that they slow down the magnetization response when the electron temperature is approaching the Curie temperature. In the LLB theory this is taken into account by the critical behavior of the longitudinal susceptibility, diverging at the Curie temperature. The effect is seen by looking at the shaded regions in Fig. 3 d) and Fig. 4. Only if the electron temperature *T_e_* stays near the Curie temperature *T_C_* for several picoseconds this transition in demagnetization behavior is found. This is a collective effect, constituting the magnetitization dynamics: from the theory of phase transitions we know that the dynamics is dominated by spin fluctuations and divergence of the correlation lengths. This leads to a slowing down of correlation times, and connected to that, a longer demagnetization time *τ_M_*^40^,^54^. The transition to the type II behavior occurs when the timescale *τ_M_*, here given in an extension valid approaching *T_C_*, exceeds the timescale *τ_E_*^22^: 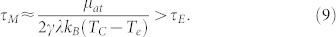
For the FePt case, a delicate balance between the driving electron temperature *T_e_*, determined by the small specific heat of the electron system for FePt, and the other parameters, drives the observation of the type II demagnetization in a small window of experimental parameters. This is in contrast to Ni, where for the same parameters only a slowing down is observed^23^.

**Figure 6:**
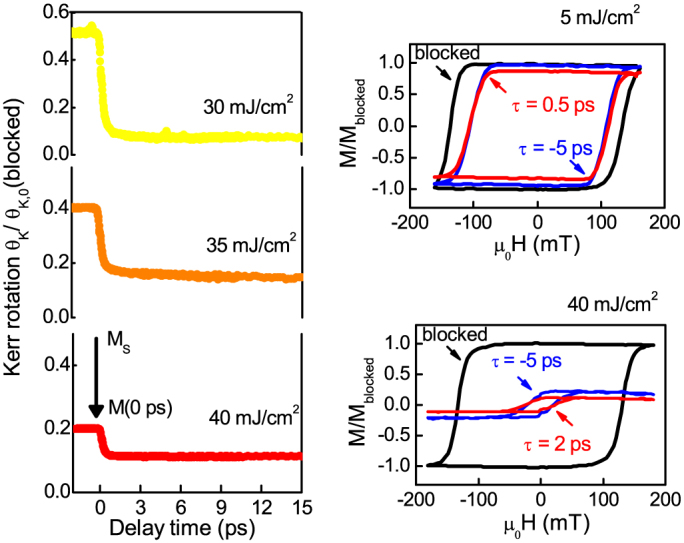
Ultrafast demagnetization dynamics experiment at highest fluences for 30 mJ/cm^2^ to 40 mJ/cm^2^. Here the time-resolved data is normalized to its magnetization value with blocked pump beam. The hysteresis curves for 5 mJ/cm^2^ and 40 mJ/cm^2^ are shown for blocked pump-beam and negative delay of −5 ps. They demonstrate the irreversible parts of the laser induced dynamics present before the new laser pulse arrives (in a time scale of 4 *μs* pulse repetition period), becoming prominent for 40 mJ/cm^2^. For 0.5 ps and 2 ps delay the hysteresis is scaled to the maximum demagnetization, demonstrating the size of the demagnetization in total (reversible and irreversible contributions).

However also certain interesting discrepancies are observed. In comparison to the model (Fig. 5), in the experiment (Fig. 2) the transition from type I to type II demagnetization takes place for a lower fluence. To investigate this further, we plot the demagnetization dynamics for the highest fluences, now normalized to the value of *M_S_* at room temperature when the pump-beam is blocked. This value can be determined by the hysteresis curves given in Fig. 6. The demagnetization relative to *M_S_* is lowered further with pump fluence and the two step behavior of the magnetization changes to a decrease within the first picosecond and stays on a plateau. This discrepancy between experiment and theory is related to the decrease of the magnetization at negative delay. The magnetization does not recover in between two laser pulses (e.g. 4 *μs* pulse repetition). These non-reversible effects are also mirrored in the strong reduction of the coercive fields, ideal for heat-assisted writing, meaning that the reversal process is significantly altered. We do not have a full understanding at the moment. Two effects could be the origin that the magnetization dynamics slows down so strongly and stays at its value. The heat is not conducted away and the repeated laser pulses increase the basis temperature, or that the magnetization itself does not recover in between two pulses completely. But it will be most interesting to explore the origin and importance for a reliable heat assisted recording technology in future hard-disc drives.

In summary, by means of time-resolved Kerr magnetometry we have investigated ultrafast magnetization dynamics in FePt thin films with perpendicular anisotropy. Our results indicate that the electron temperatures reached by the laser heating play a crucial role in the character of the ultrafast demagnetization in FePt. The measurements reveal a transition from type I to type II behavior. Our experimental results are modeled in terms of the micromagnetic LLB model, coupled to the 2T model. We discuss two scenarios with different specific heat of the electron system. Within this framework, we find that a transition to type II behavior is a consequence of the high electron temperature. We identify that at large pump fluences the resulting electron temperature remains close to the Curie temperature and is leading to critical magnetization fluctuations responsible for this transition^40^. This non-deterministic spin dynamics is responsible for a speed limitation of the magnetic response to the laser pulse. Note that this is not only determined by the ultrafast optical pulse, but also by the nature of the FePt's density of states at the Fermi level, which defines the increase in electron temperature. From our experiments we conclude that a careful adjustment between electron heating is essential, while the anisotropy energy of the system does not play a crucial role for the response time. We identified the nature of the electronic density of states as a tuning parameter for the strength, a spin system will react to the laser power. Our results open possibilities for ultrafast control of the demagnetization in FePt, the most promising candidate for future magnetic recording. Importantly, we have shown that we are able to manipulate the degree of demagnetization and its ultrafast rates. We propose that for efficient writing the degree of heating and its speed have to be balanced by varying the amount of the energy deposited.

## Methods

### Fabrication

The continuous FePt thin layer and AgCuFePt-C granular recording media have been prepared by sputtering. The structure from bottom to top is glass/NiTa/MgO/FePt/SiO_2_ and glass/NiTa/MgO/AgCuFePt-C/carbon overcoat respectively. The granular film has small amount of Ag to reduce the FePt Ll_0_ ordering temperature, and a small amount of Cu to lower the *T_C_*. Carbon intercalates the magnetic 5–8 nm grains in a specific heating cycle^32^. The continuous film sample has a Curie temperature of 650 K, a saturation magnetization *M_S_* = 1070 emu/cm^3^, a maximum anisotropy constant *K_u,max_* = 2.2 · 10^7^ erg/cm^3^, and an average anisotropy constant *K_u,av_* = 1.4 · 10^7^ erg/cm^3^. Hystersis curves are given in the Supplementary Materials and reveal for the granular recording media μ_0_ *H_S_* = 2.4 T and for the continuous FePt film μ_0_ *H_S_* = 150 mT.

### Experimental set-up

In the all-optical pump probe experiment the magneto-optical Kerr rotation of the probe beam is measured^34^. The pulse has a full width at half maximum of *τ_p_* = 40 fs and a wave length of *λ* = 800 nm (Ti:Sapphire amplifier system RegA 9040, Coherent). The repetition rate of the laser system used was 250 kHz. The Kerr rotation is extracted from that of opposite external field direction^37^. To get the absolute degree of demagnetization, the Kerr signal is scaled to hysteresis measurements at two states of reference; one at negative delay (*θ_K_* ~ *M_z_*_,0_) and the other at a time delay that shows the lowest magnetization (Δ*θ_K,min_* ~ Δ*M_z,min_*).

### Thermal modelling

To describe the ultrafast magnetization dynamics, we model a thermal process of electronic origin^23^. The model considers that within a timescale of the order of 10 fs the electrons are thermalized and can be described by a quasi-equilibrium electron temperature *T_e_*(*t*). A multi-macrospin model is used with cubic discretization elements with a lateral size of Δ = 3 nm (volume *V* = Δ^3^). Within each cell the Landau-Lifshitz-Bloch (LLB) micromagnetic formalism is applied^41^. For the present simulation, a system of 30 × 30 × 1 macrospins with periodic boundary conditions in *x* and *y* directions is used.

## Author Contributions

T.S., M.M. and O.C. designed the experiment. J.M. and J.W. carried out the experiments and analyzed experimental data. J.M. and O.C. developed the theoretical model. T.S. and S.P. prepared and characterized the samples. J.M., P.M., O.C., T.S., S.P. and M.M. discussed the data and anlysis and wrote the manuscript. All the authors reviewed the manuscript.

## Supplementary Material

Supplementary Information Supplementary Material

## Figures and Tables

**Table 1:** Overview of parameter set I for the simulation in the case of high electron temperature for FePt and set II for a hypothetic material with a large density of states at the Fermi level, the hypothetic low electronic temperature case

Parameter set	*γ_e_*	*λ*	*I*_0_	*G_e–ph_*	*C_ph_*	*τ_ph_*	*S*
	(J/m^3^K^2^)		(s^−1^)	(W/m^3^K)	(J/m^3^K)	(ps)	( 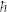 )
I	110	0.01	5.0 · 10^16^	1.5 · 10^17^	3.7 · 10^5^	340	3/2
II	1700	0.1	3.0 · 10^17^	1.8 · 10^18^	3.3 · 10^6^	340	3/2

## References

[b1] Moser A. & Weller D. in The physics of high density magnetic recording 145 (Springer, New York, 2001).

[b2] Stipe B. C. *et al.* Magnetic recording at 1.5 Pb m^2^ using an integrated plasmonic antenna. Nature Photon. 4, 484–488 (2010).

[b3] Tudosa I. *et al.* The ultimate speed of magnetic switching in granular recording media. Nature 428, 831–833 (2004). 15103370 10.1038/nature02438

[b4] Back C. *et al.* Minimum field strength in precessional magnetization reversal. Science 285, 864–867 (1999). 10436149 10.1126/science.285.5429.864

[b5] Stanciu C. D. *et al.* All-optical magnetic recording with circularly polarized light. Phys. Rev. Lett. 99, 047601 (2007). 17678404 10.1103/PhysRevLett.99.047601

[b6] Lu Z., Chepulskii R. V. & Butler W. H. First-principles study of magnetic properties of L1_0_-ordered MnPt and FePt alloys. Phys. Rev. B 81, 094437 (2010).

[b7] Barreteau C. & Spanjaard Magnetic and electronic properties of bulk and clusters of FePt L1_0_. J. Phys.: Cond. Mat. 24, 406004 (2012). 10.1088/0953-8984/24/40/406004 22987868

[b8] Djordjevic M. & Münzenberg M. Connecting the timescales in picosecond remagnetization experiments. Phys. Rev. B 75, 012404 (2007).

[b9] Kazantseva N., Nowak U., Chantrell R. W., Holhfeld J. & Rebei A. Slow recovery of the magnetisation after a sub-picosecond heat pulse. Europhys. Lett. 81, 27004 (2008).

[b10] Beaurepaire E. *et al.* Spin dynamics in CoPt_3_ alloy films: A magnetic phase transition in the femtosecond time scale. Phys. Rev. B 58, 12134 (1998).

[b11] Beaurepaire E., Merle J.-C., Daunois A. & Bigot J.-Y. Ultrafast spin dynamics in ferromagnetic nickel. Phys. Rev. Lett. 76, 4250 (1996). 10061239 10.1103/PhysRevLett.76.4250

[b12] Liu X. D. *et al.* Single laser pulse induced dynamic magnetization reversal mechanism of perpendicularly magnetized L1_0_ FePt films. J. Appl. Phys. 106, 053907 (2009).

[b13] Zhao J. Q., Cui B. Y., Zhang Z. Z., Ma B. & Jin Q. Y. Coercivity dynamics and origin of time-delayed magneto-optical hysteresis loops in pump-probe Kerr spectroscopy. Thin Solid Films 518, 2830 (2010).

[b14] Mizukami S. *et al.* Fast magnetization precession observed in FePt L1_0_-FePt epitaxial thin film. Appl. Phys. Lett. 98, 052501 (2011).

[b15] Ostler T. A. *et al.* Ultrafast heating as a sufficient stimulus for magnetization reversal in a ferrimagnet. Nature Comm. 3, 666 (2012). 10.1038/ncomms1666 22314362

[b16] Radu I. *et al.* Transient ferromagnetic-like state mediating ultrafast reversal of antiferromagnetically coupled spins. Nature 472, 205 (2011). 21451521 10.1038/nature09901

[b17] Pfau B. *et al.* Ultrafast optical demagnetization manipulates nanoscale spin structure in domain walls. Nature Comm. 3, 1100 (2012). 10.1038/ncomms2108 PMC3493637 23033076

[b18] Graves C. E. *et al.* Nanoscale spin reversal by non-local angular momentum transfer following ultrafast laser excitation in ferrimagnetic GdFeCo. Nature Mater. 12, 293298 (2013). 10.1038/nmat3597 23503010

[b19] Atxitia U. *et al.* Ultrafast dynamical path for the switching of a ferrimagnet after femtosecond heating. Phys. Rev. B 87, 224417 (2013).

[b20] Mentink J. H. *et al.* Ultrafast Spin Dynamics in Multisublattice Magnets. Phys. Rev. Lett. 108, 057202 (2012). 22400955 10.1103/PhysRevLett.108.057202

[b21] Schellekens A. J. & Koopmans B. Microscopic model for ultrafast magnetization dynamics of multisublattice magnets. Phys. Rev. B 87, 020407(R) (2013).

[b22] Koopmans B. *et al.* Explaining the paradoxical diversity of ultrafast laser-induced demagnetization. Nature Mater. 9, 259 (2010). 20010830 10.1038/nmat2593

[b23] Atxitia U., Chubykalo-Fesenko O., Walowski J., Mann A. & Münzenberg M. Evidence for thermal mechanisms in laser-induced femtosecond spin dynamics. Phys. Rev. B 81, 174401 (2010).

[b24] Sultan M., Atxitia U., Melnikov A., Chubykalo-Fesenko O. & Bovensiepen U. Electron-and phonon-mediated ultrafast magnetization dynamics of Gd(0001). Phys. Rev. B 85, 184407 (2012).

[b25] Roth T. *et al.* Temperature dependence of laser-induced demagnetization in Ni: A key for identifying the underlying mechanism. Phys. Rev. X 2, 021006 (2012).

[b26] Battiato M., Carva K. & Oppeneer P. M. Superdiffusive spin transport as a mechanism of ultrafast demagnetization. Phys. Rev. Lett. 105, 027203 (2010). 20867735 10.1103/PhysRevLett.105.027203

[b27] Rudolf D. *et al.* Ultrafast magnetization enhancement in metallic multilayers driven by superdiffusive spin current. Nature Comm. 3, 1037 (2012). 10.1038/ncomms2029 22948819

[b28] Melnikov A. *et al.* Ultrafast transport of laser-excited carriers in Au/Fe/MgO(001). Phys. Rev. Lett. 107, 076601 (2011). 21902412 10.1103/PhysRevLett.107.076601

[b29] Kampfrath T. *et al.* Terahertz spin current pulses controlled by magnetic heterostructures. Nature Nanotech. 8, 256–260 (2013). 10.1038/nnano.2013.43 23542903

[b30] Kazantseva N. *et al.* Towards multiscale modeling of magnetic materials: Simulations of FePt. Phys. Rev. B 77, 184428 (2008).

[b31] Müller G. *et al.* Spin polarization in half-metals probed by femtosecond spin excitation. Nature Mater. 8, 56–61 (2009). 19079243 10.1038/nmat2341

[b32] Mosendz O., Pisana S., Reiner J. W., Stipe B. & Weller D. Ultra-high coercivity small-grain FePt media for thermally assisted recording. J. Appl. Phys. 111, 07B729 (2012).

[b33] McCallum A. T., Kercher D., Lille J., Weller D. & Hellwig O. Prevention of dewetting during annealing of FePt films for bit patterned media applications. Appl. Phys. Lett. 101, 092402 (2012).

[b34] Walowski J. *et al.* Intrinsic and non-local Gilbert damping in polycrystalline nickel studied by Ti:sapphire laser fs spectroscopy. J. Phys. D: Appl. Phys. 41, 164016 (2008).

[b35] Atxitia U. & Chubykalo-Fesenko O. Ultrafast magnetization dynamics rates within the Landau-Lifshitz-Bloch model. Phys. Rev. B 84, 144414 (2011).

[b36] Carpene E. *et al.* Measurement of the magneto-optical response of Fe and *CrO*_2_ epitaxial films by pump-probe spectroscopy: Evidence for a spin-charge separation. Phys. Rev. B 87 (2013).

[b37] Kampfrath T. *et al.* Ultrafast magneto-optical response of iron thin films. Phys. Rev. B 65, 104429 (2002).

[b38] Bigot J. Y. *et al.* Coherent ultrafast magnetism induced by femtosecond laser pulses. Nature Phys. 5, 515–520 (2009).

[b39] Garanin D. A. Generalized equation of motion for a ferromagnet. Physica A 172, 470–491 (1991).

[b40] Chubykalo-Fesenko O., Nowak U., Chantrell R. W. & Garanin D. Dynamic approach for micromagnetics close to the Curie temperature. Phys. Rev. B 74, 094436 (2006).

[b41] Atxitia U. *et al.* Micromagnetic modeling of laser-induced magnetization dynamics using the Landau-Lifshitz-Bloch equation. Appl. Phys. Lett. 91, 232507 (2007).

[b42] Garanin D. & Chubykalo-Fesenko O. Thermal fluctuations and longitudinal relaxation of single-domain magnetic particles at elevated temperatures. Phys. Rev. B 70, 212409 (2004).

[b43] Lyberatos A. & Guslienko K. Y. Thermal stability of the magnetization following thermomagnetic writing in perpendicular media. J. Appl. Phys. 94, 1119–1129 (2003).

[b44] Okamoto S. *et al.* Chemical-order-dependent magnetic anisotropy and exchange stiffness constant of FePt (001) epitaxial films. Phys. Rev. B 66, 024413 (2002).

[b45] Mryasov O. N., Nowak U., Guslienko K. Y. & Chantrell R. W. Temperature-dependent magnetic properties of FePt: Eective spin Hamiltonian model. Europhys. Lett. 69, 805–811 (2005).

[b46] Atxitia U. *et al.* Multiscale modeling of magnetic materials: Temperature dependence of exchange stiffness. Phys. Rev. B 82, 134440 (2010).

[b47] Evans R. F. L. *et al.* Stochastic form of the Landau-Lifshitz-Bloch equation. Phys. Rev. B 85, 014433 (2012).

[b48] Caffrey A. P., Hopkins P. E., Klopf J. M. & Norris P. M. in Thin film non-noble transition metal thermophysical properties, 365–377 (Taylor & Francis Inc., 2005).

[b49] Chen Z. *et al.* Spin waves and small intrinsic damping in an in-plane magnetized FePt film. Appl. Phys. Lett. 101, 222402 (2012).

[b50] Mizukami S. *et al.* Fast magnetization precession observed in L1_0_-FePt epitaxial thin film. Appl. Phys. Lett. 98, 052501 (2011).

[b51] He P. *et al.* Quadratic scaling of intrinsic Gilbert damping with spin-orbital coupling in L1_0_-FePdPt films: experiments and ab initio calculations. Phys. Rev. Lett. 110, 077203 (2013). 10.1103/PhysRevLett.110.077203 25166400

[b52] Mo N. *et al.* Origins of the damping in perpendicular media: Three component ferromagnetic resonance linewidth in CoCrPt alloy films. Appl. Phys. Lett. 92, 022506 (2008).

[b53] Hohlfeld J. *et al.* Electron and lattice dynamics following optical excitation of metals. Chem. Phys. 251, 237–258 (2000).

[b54] Fisher D. Scaling and critical slowing down in random-field Ising systems. Phys. Rev. Lett. 56, 416–419 (1986). 10033187 10.1103/PhysRevLett.56.416

