# Supplementary material for: Resolving the role of femtosecond heated electrons in ultrafast spin dynamics
Source: Sci Rep. 2014 Feb 5;4:3980. doi: 10.1038/srep03980 (PMC3913971; doi:10.1038/srep03980)
Supplement: Supplementary Information — Supplementary Material [file srep03980-s1.pdf]

Supplementary Materials: Resolving the role of femtosecond heated electrons in ultrafast spin dynamics

J. Mendil,[1] P. Nieves,[2] O. Chubykalo-Fesenko,[2] J. Walowski,[1] T. Santos,[3] S. Pisana,[3] and M. Münzenberg[1, a)]

[1)] *I. Physikalisches Institut, Universität Göttingen, Friedrich-Hund Platz 1, 37077 Göttingen, Germany*

[2)] *Instituto de Ciencia de Materiales de Madrid, CSIC, Cantoblanco, 28049 Madrid, Spain*

[3)] *San Jose Research Center, HGST, a Western Digital Company, 3403 Yerba Buena Rd., San Jose, California 95135, USA*

(Dated: 18 December 2013)

[a)] Electronic mail: mmuenze@gwdg.de

Parameter set	γ_e	λ	I_0	G_{e-ph}	C_{ph}	τ_{ph}	S
	(J/m^{3}K^2)		(s^{-1})	(W/m^{3}K)	(Jm^{-3}K^{-3})	(ps)	($\hbar$)
I	110	0.01	$5.0 \cdot 10^{16}$	$1.5 \cdot 10^{17}$	$3.7 \cdot 10^5$	340	3/2
II	1700	0.1	$3.0 \cdot 10^{17}$	$1.8 \cdot 10^{18}$	$3.3 \cdot 10^6$	340	3/2

TABLE I. Overview of parameter set I for the simulation in the case of high electron temperature and set II for the hypothetic low electronic temperature case.

I. DETAILED SIMULATIONS FOR SCENARIO II

We present the detailed results of the simulations corresponding to the second set of the parameters refereed in the main text, which results in a low electron temperature.

In Fig. 1 we show the electronic temperature dynamics using the 2TM for the second hypothetic scenario. The values are chosen such that the same relaxation time τ_E is reached, however the parameter γ_e that defines the electron specific heat is strongly increased. The other parameters are matched to get the correct dynamics mirrored in relaxation time τ_E. In addition, the condition was that the maximum demagnetization should be similar in scenario II. As a consequence of the increased γ_e, in the second scenario the maximum electron temperature is up to 600 K smaller than in the high electronic temperature case presented in the main manuscript. For comparison with the experimental values, in Fig. 2 we present the relaxation time τ_E of the electronic temperature using the 2TM for different values of fluence. The experimental and theoretical values for τ_E are in relative agreement.

In Fig. 3 we present the magnetization dynamics using the integration of the stochastic LLB equation, coupled with the 2T model for this low electron temperature case. Because of the lower electron temperatures, we need large values of $\lambda = 0.1$ to get similar values of $\triangle M/M_{300K}$ and τ_M. For other reasonable values of $\lambda \in [0.01, 0.1]$[1] we obtain values of $\triangle M/M_{300K}$ and τ_M that not large enough compared to the experiments. As it can be seen, no transition to the type II behavior is reproduced. As a result, the demagnetization time τ_M is almost constant in the simulations and the values are different from the one that is obtained in the experiment (Fig. 2). Even though a very good quantitative agreement of the relaxation time τ_E is given, because of the fact that the electron temperature exceeds

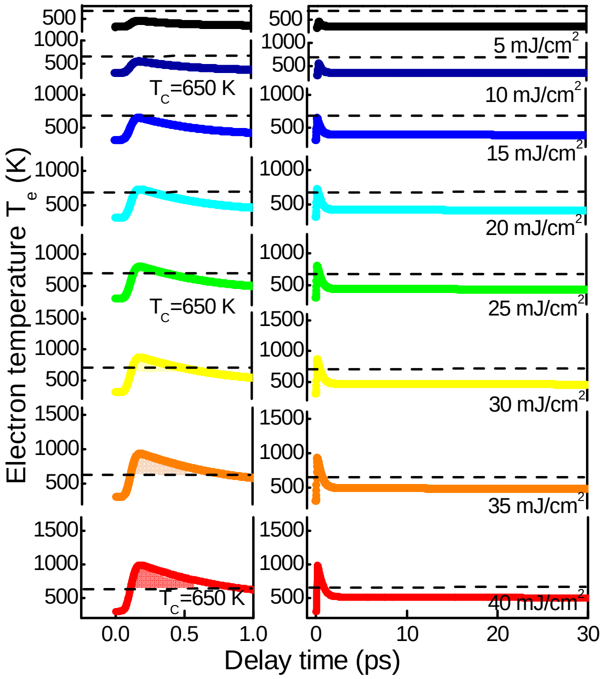

FIG. 1. Simulation of the electron temperature T_e, shown as a function of the laser pump fluence (from 5 mJ/cm^2 (upper curve) to 40 mJ/cm^2 (lower curve), in steps of 5 mJ/cm^2. The 2T model is based on the parameter set II. Within the shaded area marked in the left panel, the electron temperature exceeds the Curie temperature (dashed line). This shaded area is very small for parameter set II.

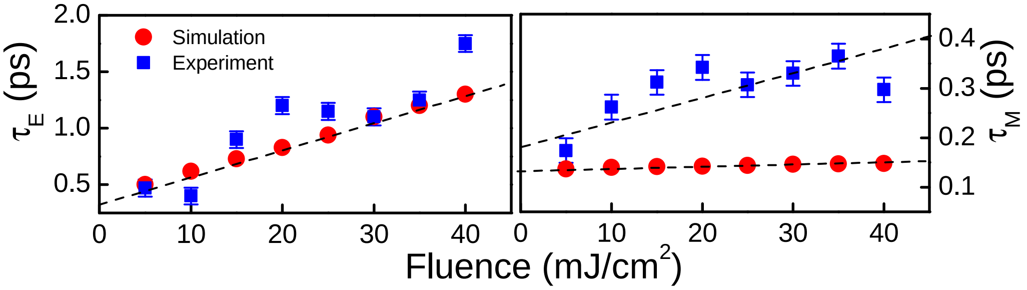

FIG. 2. Results for the relaxation time τ_E of the electronic temperature using the 2TM and the demagnetization time τ_M extracted from the model using parameter set II. The dashed line marks the linear increase.

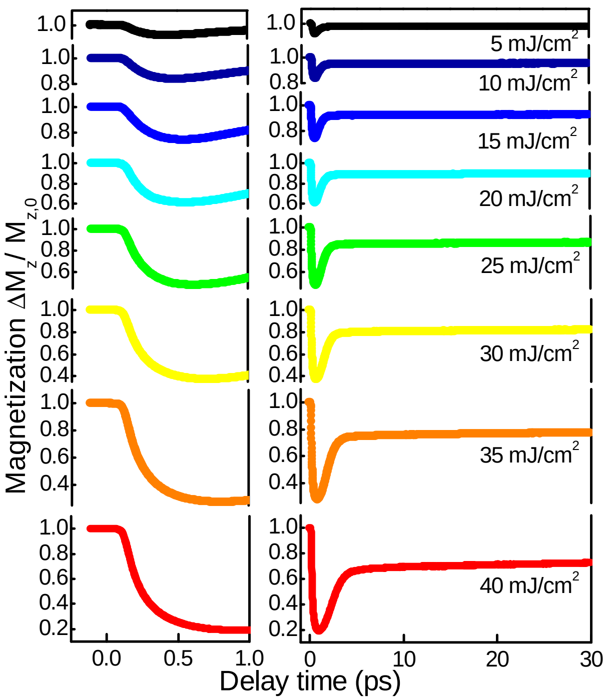

FIG. 3. Numerical results of the magnetization dynamics using the stochastic LLB equation for a multi-macrospin model and using the value of the parameters presented in Table I, parameter set II.

the Curie temperature only very little and for a short time (Fig. 1), no increase of the demagnetization time τ_M is observed.

II. HYSTERSIS CURVES

The hysteresis curve reveals a saturation field of 150 mT for the FePt film. In the experimental setup, where the time-resolved experiments are conducted, a field of $+/-$ 200 mT (max. $+/-$ 350 mT available) was applied to saturate the samples out-of-plane. The granular films with saturation field of 2.4 T were saturated by a $+/-$ 5T field prior to the experiment in the remanent state and the laser fluence was kept in the few degree demagnetization to reestablish 100%M_S after each laser shot. The time-resolved data for both magnetization directions $+/-M_z$ were measured in parallel on identical samples. As another outcome of the performed Kerr-magnetometry, we observe an offset demagnetization before each pump pulse. The offset demagnetization increases with growing fluence. It is measured via hysteresis loops at a delay time before a pump pulse ($\tau = -5$ ps). Scaled

to the hysteresis loop at blocked pump beam, and thus at the sample being thermalized to room temperature, a decrease of the saturation magnetization M_s with increasing fluence is evident (see hysteresis loops in Fig. 6, main text). The heat accumulation is arising from a low heat conduction of the substrate (glass) could lead to a remaining elevation of temperature before the next pump pulse arrives. This leads for highest fluence to a decrease of the coercive field H_C with increasing fluence. This characteristic is desirable for heat-assisted magnetic recording applications. The coercive field H_C decreases to 0.2 approaching high fluences of 40 mJ/cm^2. We want to remark that the coercive field is a macroscopic variable, whose properties are determine, for example by pinning and other irreversible process and does not necessarily allow deduction of microscopic processes of the ultrafast demagnetization. This strong demagnetization, already present at negative delay, is the origin of the discrepancy of the experimental spectrum (Fig. 2, main manuscipt) and simulated dynamics (Fig. 5, main manuscipt), starting to deviate above 35 mJ/cm^2 at the highest fluences measured. The hysteresis curves for the different delay are included for a direct comparison with the time-resoved data in Fig. 6, main manuscipt.

REFERENCES

[1]Kazantseva, N. et al., Towards multiscale modeling of magnetic materials: Simulations of FePt, *Phys. Rev. B* **77**, 184428 (2008).
